# Evaluation of a Polymerase Chain Reaction Assay for Pathogen Detection in Septic Patients under Routine Condition: An Observational Study

**DOI:** 10.1371/journal.pone.0046003

**Published:** 2012-09-27

**Authors:** Frank Bloos, Svea Sachse, Andreas Kortgen, Mathias W. Pletz, Marc Lehmann, Eberhard Straube, Niels C. Riedemann, Konrad Reinhart, Michael Bauer

**Affiliations:** 1 Deptartment of Anesthesiology and Intensive Care Therapy, Jena University Hospital, Jena, Germany; 2 Center for Sepsis Control & Care, Jena University Hospital, Jena, Germany; 3 Jena University Hospital, Institute of Medical Microbiology, Jena, Germany; 4 Divison of Gastroenterology, Hepatology and Infectious Diseases, Jena University Hospital, Jena, Germany; 5 SIRS-Lab GmbH, Jena, Germany; D'or Institute of Research and Education, Brazil

## Abstract

**Background:**

Treatment of septic shock relies on appropriate antimicrobial therapy. Current culture based methods deliver final results after days, which may delay potentially lifesaving adjustments in antimicrobial therapy. This study was undertaken to compare PCR with blood culture results under routine conditions regarding 1. impact on antimicrobial therapy, and 2. time to result, in patients with presumed sepsis.

**Methodology/Principal Findings:**

This was an observational study in a 50 beds ICU of a university hospital. In 245 patients with suspected sepsis, 311 concomitant blood cultures and blood for multiplex PCR (VYOO®) were obtained. 45 of 311 blood cultures (14.5%) and 94 of 311 PCRs (30.1%) were positive. However, blood culture or microbiological sampling from the presumed site of infection rarely confirmed PCR results and vice versa. Median time to positivity and interquartile range were 24.2 (18.0, 27.5) hours for the PCR and 68 (52.2, 88.5) hours for BC (p<0.01). PCR median time to result was dependent on technician availability (53.5 hours on Saturdays, 7.2 hours under optimal logistic conditions). PCR results showed good correlation with procalcitonin (p<0.001). In 34% of patients with positive PCRs antimicrobial therapy was considered inadequate according to assessment of clinical arbitrators including 5 patients with vancomycin-resistant enterococci (VRE), 3 cases with multiresistant staphylococci, and 4 patients with fungi.

**Conclusions:**

The results of this observational study support the hypothesis that PCR results are available faster, are more frequently positive, and may result in earlier adjustment of antimicrobial therapy. However, shorter time to result can only be fully exploited when the laboratory is adequately staffed for a 24 hour/7 day service, or when point of care/automated assay systems become available.

## Introduction

Sepsis is a leading cause of death in hospitalized patients worldwide, with a continuous increase in incidence of approximately 5–10% per year [1]. Fast and adequate antimicrobial therapy is crucial in these patients, and clinical studies have unequivocally shown that inappropriate initial treatment results in up to a five-fold increase in mortality; this increase is most dramatic in patients with septic shock [2], [3]. Reasons for inappropriate treatment include i) failure to correctly cover the underlying pathogen, ii) choosing an anti-infective with poor penetration into the infectious focus, and iii) underdosing. Whereas all three reasons may be equally important, PCR-based diagnostics could reduce the share of failed coverage [4].

The current guideline-driven strategy for empirical antimicrobial therapy of underlying pathogens is broad spectrum antibiotics with anti-pseudomonal acitivity [5]. The disadvantage of this strategy is the increasing selection of antibiotic resistant pathogens. Nevertheless, for the individual patient even this guideline-driven empiric broad approach might be insufficient in three scenarios: infections by fungi, by multi-resistant bacteria and, less frequently parasitic and viral infections.

Faster microbiological workup could reduce the possibly fatal consequences of inappropriate antimicrobial therapy [6]. Conventional detection of underlying pathogens relies on culture-based methods such as blood culture or microbiological specimens from the presumed site of infection. However, results from these techniques are frequently negative, even in septic shock, or are only available after several days, which leaves correct antimicrobial therapy to chance. Furthermore, microbiological samples are very sensitive to errors in the pre-analytical handling of the specimen (transport, medium, storage) [7].

Culture-independent molecular biology based techniques, such as Polymerase Chain Reaction (PCR), may offer a solution to the problems associated with culture-dependent methods. Recent studies from various groups with a commercially available multiplex PCR assay for pathogen detection were promising, with the rate of positivity being about two-fold higher than with blood culture [8]–[10]. It was also concluded that PCR may be a helpful add-on in patients with infection where blood cultures remained negative [11], in particular since recent data applying mathematical modelling demonstrated that PCR-dependent pathogen detection may be cost-effective despite the high direct costs of PCR [12]. However, these conclusions are hypothetical, since PCR was not yet tested in a clinical setting where physicians include PCR results in their treatment decisions.

The primary objective of the current study was to compare the performance of PCR to detect microbial DNA with pathogen detection by blood culture and conventional microbiological results in patients with presumed sepsis in daily clinical practice. Data regarding the impact of PCR-based microbial detection on the clinical course of patients are very limited. In one prospective randomized study, patients who had received allogeneic stem-cell transplantation showed a lower 30 day mortality rate when treated with liposomal amphotericin B according to a PCR-based algorithm than when treated empirically [13]. Such data are missing in non-neutropenic critically ill patients. The second goal of this study was to assess the degree to which PCR results were associated with changes to the pre-existing antimicrobial therapy.

In principle, PCR technology currently delivers results within 6 to 8 hours. However, such a short time requires optimal logistic and technical conditions and has never been challenged in the routine clinical setting. A further goal of this study was thus to compare the turn-around times of PCR and blood culture from blood sampling until release of final result to the treating physician.

## Methods

### Ethics statement

The study was approved by the institutional ethics committee of the Friedrich-Schiller-University Jena, which waived the need for informed consent due to the observational nature of the study. Patient data were de-identified by consecutive numbers and analysed anonymously. Only study personnel bound to confidentiality were able to reidentify data entries for quality control.

### Patients

This study was an observational trial comparing the results of PCR with blood culture and microbiological results from the presumed source of sepsis. In critically ill patients treated on an interdisciplinary ICU between May 2009 and June 2010, blood for PCR was obtained in parallel if a blood culture was taken for suspected sepsis. Patients less than 18 years old were excluded from the study.

### Procedures

20 ml blood were taken by sterile venous puncture and distributed equally for conventional blood cultures into aerobic and anaerobic media by the treating physician. In addition to each pair of cultures, 10 ml of EDTA blood were taken for PCR analysis. Consecutive samples were taken if further blood cultures were deemed necessary due to the clinical course of the patient.

The multiplex PCR-based assay used (VYOO®, SIRS-Lab GmbH, Jena) combines a bead-based mechanical lysis protocol, a DNA preparation that includes the separation and relative enrichment of microbial DNA from background human DNA, and a multiplex PCR protocol [14]. The version of the assay that was used identified amplicons by electrophoresis, and detected DNA from a panel of 34 bacterial and 6 fungal species known to cause sepsis, as well as five genes coding for important antibiotic resistances (mecA: methicillin; vanA and vanB: vancomycin; several variants of blaSHV and blaCTX: extended spectrum β-lactamase).

Detailed procedures of the sample preparation for the VYOO® assay have been described in detail elsewhere [14]. Briefly, 5 ml EDTA blood were mechanically lysed in a bead-beating device (FastPrep®-24, MP Biomedicals, Illkirch, France) over two periods of 45 s. For further DNA extraction, lysate treated with proteinase-K and lysis buffer at 50°C over 30 minutes was processed immediately afterwards, according to the kit instructions. The DNA pellet was re-suspended in 300 µl of sample buffer. For depletion of human DNA and concomitant enrichment of microbial DNA, total DNA was applied to an affinity chromatography column with an immobilized, truncated human CpG-binding protein motif (LOOXSTER®, SIRS-Lab, Jena, Germany). After binding and rinsing, concentrated microbial DNA was eluted, precipitated with isopropanol/3 M NaAc pH 4.6, washed with ice-cold 70% ethanol, air dried and re-suspended in 30 µl nuclease-free water. The DNA concentration was determined by spectrophotometric measurement at 260 nm with a NanoDrop® ND-1000 (Nanodrop Technologies, Wilmington, USA). Finally, DNA was adjusted to 1 µg per 25 µl multiplex- PCR reaction and applied to two primer pools. The amplification was carried out in a Mastercycler® ep gradient S (Eppendorf, Hamburg, Germany) under the following conditions: initial denaturation at 95°C for 15 min, a cycling programme consisting of 30 three-step cycles of 30 s at 94°C, 90 s at 59°C and 45 s at 72°C, with a terminal extension step at 72°C for 10 min. Sizes of amplicons were analysed in ethidium bromide-stained 3% agarose gels, and compared with defined molecular marker bands provided in the kit. Inhibitions in negative samples could be excluded by generation of positive inhibition controls performed for all DNA samples.

Pairs of BCs were incubated at 37°C and monitored for 8 days. Isolated micro-organisms and their susceptibilities were determined by standard methods and criteria [15]. Blood culture and PCR results were reported to the physician in charge as soon as the results were available.

### Collection of additional data

The host response was monitored in all patients by leukocyte count, procalcitonin (PCT), and C-reactive protein (CRP). Results of all microbiological specimens obtained three days before and after the PCR sample were analysed and correlated with BC and PCR results. An independent infectious diseases consultant and an ICU consultant served as clinical arbitrators and reviewed appropriateness of antimicrobial therapy in patients with a positive PCR and assessed the impact of this result. The classification of Cohen and Calandra was used to define microbiologically confirmed, probable and possible infection [16].

### Statistical methods

Data are considered to be not normally distributed. Therefore, discrete variables are expressed as percentages and continuous variables as median, 25th and 75th percentiles, unless stated otherwise. Frequencies were compared by the chi-square test, and differences of medians by the Mann-Whitney test. Biomarker plasma levels according to blood culture and PCR results were compared by the Kruskal-Wallis rank sum test. The Mann-Whitney test with Bonferroni correction was applied as a post-hoc test when the Kruskal-Wallis test delivered a significant result. Duration until positive PCR or blood culture was analysed as a time to event analysis by using Kaplan-Meier Curves. The Log-test served to detect differences between curves. Data processing and statistical analysis was done with R version 2.10.0 [17]. A two-sided p of less than 0.05 was considered to be statistically significant.

## Results

### Demographics, characteristics, indicators of morbidity and mortality

245 patients were included into this study, all of whom were treated for presumed sepsis. 336 blood samples for PCR were obtained from these patients. For 25 of the PCRs, the concomitant blood was not obtained or did not reach the laboratory, resulting in 311 complete pairs of blood culture with concomitant PCR. Demographic data are shown in table 1. The study population consisted mainly of patients who had undergone general or heart/thoracic surgery. 84 of the patients (34.3%) died on the ICU. In 25 patients, infection was confirmed by conventional microbiological methods, while infection was assessed as clinically likely in 159 patients, and as possible in 10 patients; the source of infection remained unknown in 51 patients. Site of infections were as follows: intra-abdominal infections (30%), pneumonia (29.7%), trachea-bronchitis (15.1%), catheter-related (8.6%), wound-infections (6.6%), primary bacteremia (5.5%), cardiovascular (4.0%), other site of infection, and unknown site of infection (3.4%).

**Table 1:** Demographic data.

	Survivor	Non-survivor	All	p-value
	(N = 161)	(N = 84)	(N = 245)	
**Male gender**	60.9% (98)	63.1% (53)	61.6% (151)	0.734
**Age [years]**	67.0 (55.0; 74.0)	67.5 (55.0; 74.0)	67.0 (55.0; 74.0)	0.791
**BMI [kg/m^2^]**	26.2 (23.5; 30.5)	25.4 (23.4; 28.7)	26.1 (23.4; 30.1)	0.100
**Type of surgery**				0.289
General surgery	44.1% (71)	46.4% (39)	44.9% (110)	
Heart/thoracic surgery	29.2% (47)	32.1% (27)	30.2% (74)	
Neurosurgery	11.8% (19)	6.0% (5)	9.8% (24)	
Other	14.9% (24)	15.5% (13)	15.1% (37)	
**Charlson Comorbidity Index**	2 (1; 3)	1 (3; 4)	2 (1; 4)	0.130
**Apache II**	17.0 (12.0; 21.0)	20.5 (15.8; 26.2)	18.0 (13.0; 23.0)	<0.001
**SAPS II**	38.0 (31.0; 46.0)	50.0 (40.8; 58.2)	42.0 (33.0; 52.0)	<0.001
**ICU LOS [days]**	8.0 (3.0; 19.0)	12.0 (4.0; 24.2)	9.0 (3.0; 21.0)	0.119
**Hospital LOS [days]**	32.5 (21.8; 50.2)	20.0 (12.8; 35.0)	28.0 (17.0; 46.0)	<0.001

Data are given as frequencies or medians (25^th^; 75^th^ percentiles); p-values describe statistical differences between survivors and non-survivors; BMI: Body Mass Index; SAPSII: Simplified Acute Physiology score; LOS: length of stay.

### Culture results and corresponding culture-independent pathogen detection

311 pairs of blood cultures and PCRs were obtained. 45 (14.5%) of the blood cultures (BC) were positive, compared with 94 (30.2%) positive PCRs (p<0.001). 27 (8.7%) samples showed a concordantly positive result in both tests, while 199 (64.0%) samples were negative in both tests. There were 67 (21.5%) positive PCRs despite negative BC, while 18 (5.8%) PCRs remained negative despite a positive BC. Thus, sensitivity of the PCR to detect culture positive bacteremia was 0.6 (95% confidence interval: 0.44–0.74) and a corresponding specificity of 0.75 (0.69–0.8).

Table 2 lists the pathogens detected in the PCR and the blood culture. 40% of pathogens detected in the blood culture have also been found in the PCR (recovery rate). 30.6% of the pathogens detected in the BC were coagulase-negative staphylococci (CoNS), compared with 17.2% in the PCR. Recovery rate without CoNS was 44.4%.

**Table 2:** Microorganisms detected by blood culture and PCR.

Pathogen	BC	PCR	recovery rate
*Enterococcus faecium*	16.0% (8)	20.7% (24)	5
*Escherichia coli*	6.0% (3)	19.8% (23)	3
CoNS	30.6% (15)	17.2% (20)	4
*Staphylococcus aureus*	14.0% (7)	10.3% (12)	4
*Streptococcus bovis*	0	5.2% (6)	–
*Enterococcus faecalis*	4.0% (2)	4.3% (5)	1
*Pseudomonas aeruginosa*	2.0% (1)	4.3% (5)	1
*Morganella morganii*	0	4.3% (5)	–
*Candida* spp.*	12.0% (6)	4.3% (5)	1
*Klebsiella spp.*	2.0% (1)	4.3% (5)	1
*Stenotrophomonas maltophilia*	0	1.7% (2)	–
*Aspergillus fumigatus*	0	0.9% (1)	–
*Bacteroides fragilis*	0	0.9% (1)	–
*Prevotella buccae*	0	0.9% (1)	–
*Proteus mirabilis*	0	0.9% (1)	–
Other Gram negative bacteria**	6.0% (3)	0	0
Other Gram positive bacteria**	6.0% (3)	0	0

Recovery rate shows number of microorganisms of the concomitant blood culture also detected by the PCR. Only blood cultures with a concomitant PCR and vice versa are enlisted. Overall recovery rate is 40%. CoNS: Coagulase negative staphylococci.

* : The assay detects a conserved gene contained by all fungi, reported as ‘pan-fungi’ without further classification of the species.

** : Pathogens not on the VYOO® target list; Gram negative: bacteroides ovatus: 2, enterococcus avium: 1; Gram positive: unspecified cocci: 2, streptococcus infantarius: 1.

**Figure 1:**
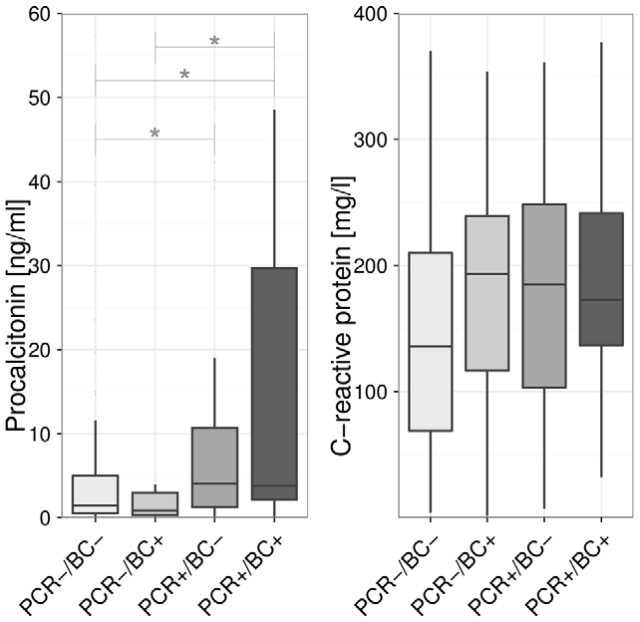
C-reactive protein (CRP) and procalcitonin (PCT) depending on blood culture (BC) and PCR result. PCR−/BC−: n = 199, PCR−/BC+: n = 18, PCR+/BC−: n = 67. PCR+/BC+: n = 27. Pathogens in the blood culture in the ‘PCR− BC+’ subgroup: CoNS: 8, *Candida albicans*: 5, *Staphylococcus aureus*: 3, *Enterococcus faecium*: 2, *Enterococcus faecalis*: 1. Kruskal-Wallis for PCT: p<0.001; *: p<0.008 (Mann-Whitney test, Bonferroni correction for multiple testing). Kruskal-Wallis-Test for CRP: p = 0.01; no significant differences between groups were detected.

PCR positive patients had higher procalcitonin (PCT) levels than PCR negative and blood culture negative patients (figure 1). Patients with negative blood culture and negative PCR had also a lower C-reactive protein (CRP) than patients with either positive PCR or positive blood culture (Kruskal-Wallis test: p = 0.01). However, the post-hoc test failed to identify statistical significant differences between specific groups after correcting for multiple testing. White blood cell counts did not differ between positive and negative PCRs, nor between positive and negative BCs. Neither the result of the PCR nor the result of the BC was associated with ICU mortality or a difference in the SOFA-score.

On the sampling day, patients positive for CoNS in the BC had median CRP levels of 156 (107; 204) mg/l and PCT levels of 1.4 (0.7; 6.0) ng/ml. These were not different from the levels of CRP 168 (114; 217, p = 0.794) mg/l and PCT 2.9 (1.6; 8.0; p = 0.397) ng/ml on days with positive PCRs for CoNS. Presence of a fungal amplicon compared to presence of a bacterial amplicon in the PCR was not associated with differences in leukocyte count, CRP, or PCT.

### Confirmation of PCR at onset of sepsis by microbiological specimen

**Figure 2:**
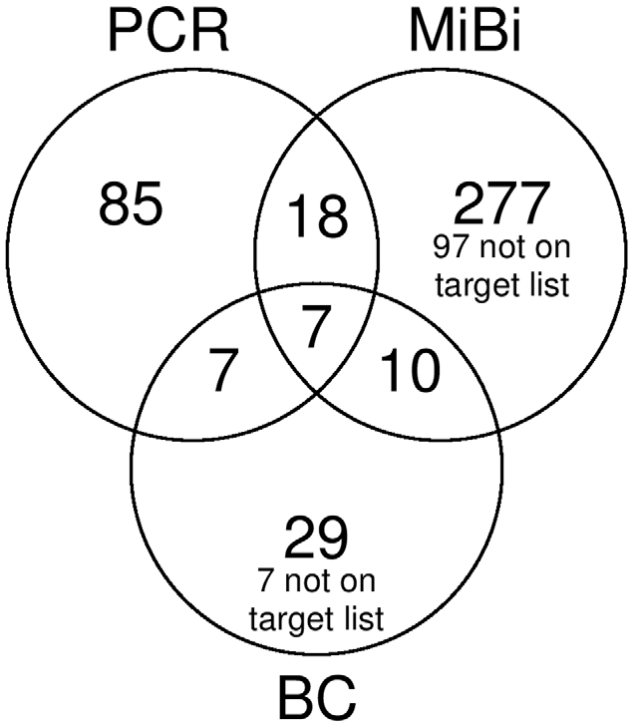
Concordance of detected microorganism between PCR and culture based methods. Venn diagram showing the number of microorganisms found in the PCR, the concomitant blood culture, and the microbiological specimens from the presumed site of infection. Numbers in the overlapping areas reflect microorganisms detected with more than one technique simultaneously. Most of the pathogens were not confirmed with any other technique. Unconfirmed results from the culture based methods are partly not on the PCR target list. Results where the same pathogen was detected more than once in the same specimen were removed from this diagram.

Irrespective of the BC taken concomitantly with the PCR, 312 pathogens were detected by the microbiological sample taken from the presumed site of infection (figure 2). 7.9% of these microorganisms were confirmed by PCR from blood (table 3). The blood culture similarly confirmed 17 (5.4%) pathogens. 7 of the pathogens detected by BC were not included in the panel of the PCR, i.e. *Bacteroides ovatus*, *Enterococcus avium*, and several streptococcal species (figure 2). 277 pathogens taken from the presumed site of infection were not found by either blood culture or PCR. 85 of the pathogens detected by PCR were not found by blood culture or microbiological sampling from the presumed site of infection.

**Table 3:** Microorganisms detected in microbiological samples from presumed site of infection compared to PCR.

Pathogen	BC	recovery rate
*CoNS*	15.6% (39)	1
*Candida albicans*	10.7% (30)	1
*Escherichia coli*	9.3% (26)	5
*Pseudomonas aeruginosa*	7.5% (21)	0
*Staphylococcus aureus*	6.8% (19)	5
Yeasts (not further specified)**	6.0% (19)	0
*Klebsiella spp.*	5.0% (14)	3
*Enterococcus faecium*	4.3% (12)	2
*Non-albicans Candida spp.*	3.9% (11)	1
*Enterobacter cloacae*	3.6% (10)	0
*Enterococcus faecalis*	3.6% (10)	2
*Stenotrophomonas maltophilia*	3.2% (9)	0
*Corynebacteria* **	3.2% (9)	0
*Serratia marcescens*	2.1% (6)	1
*Morganella morganii*	1.8% (5)	0
*Proteus mirabilis*	1.4% (4)	1
*Streptococcus pneumoniae*	0.7% (2)	0
*Burkholderia cepacia*	0.7% (2)	0
*Aspergillus fumigatus*	0.4% (1)	0
*Haemophilus influenzae*	0.4% (1)	0
Other Gram positive bacteria**	6.4% (16)	0
Other Gram negative bacteria**	4.6% (13)	0
Other fungi**	1.8% (5)	0

Recovery rate shows number of microorganisms of the microbiological samples from presumed site of infection within 3 days before and after the PCR sampling also detected by the PCR. Same pathogens found in different samples of the same patient are only counted once. The overall recovery rate is 7.9%. CoNS: Coagulase negative staphylococci.

* : The assay detects a conserved gene contained by all fungi, reported as ‘pan-fungi’ without further classification of the species.

** : Pathogens not on the VYOO® target list.

### Time to result – comparison of PCR and BC

**Figure 3:**
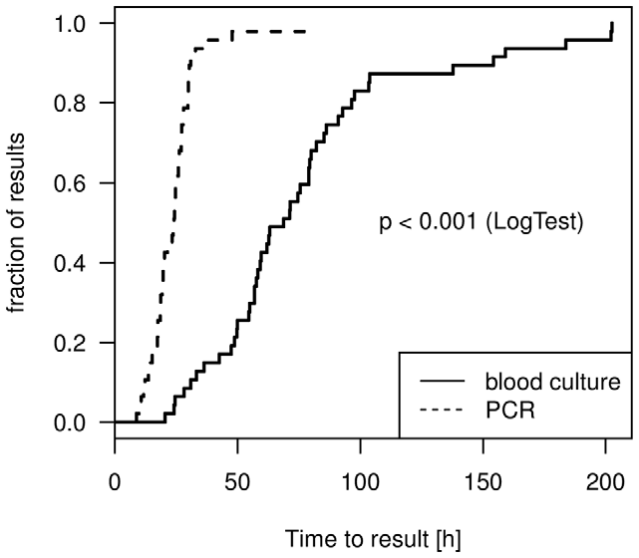
Time to positive result for PCR and blood culture (Kaplan-Meier-Curve). Blood culture and PCR, which remain negative, have been excluded from this analysis. Median time to result was 25.2 (interquartile range: 18.2–32.2) hours for the PCR versus 189.5 (178.6–197.4) hours for the blood culture.

Median time to positivity was significantly shorter for PCR (24.2 [interquartile range 18.0–27.5] hours) than for blood culture (68 [52.2–88.5] hours, p<0.001) (fig. 3). Time to positivity in PCRs was not statistically different from time to negative PCR results (25.1 [18.1–32.2] hours, p = 0.422). Time to final result including negative test results were 189.5 (178.6–197.4) hours for blood culture and 25.2 (18.2–32.3, p<0.001) hours for PCR. The longest median times to result for PCRs were observed on Saturdays, with 53.5 (45.4; 64.0) hours compared with 23.4 (16.7; 29.2) hours during the week; this was primarily due to the lack of technician support for conducting the PCR at weekends. When all technical requirements were optimized, positive PCR results could be obtained in 7.2 hours after blood sampling. Time to blood culture result was significantly lower in positive BCs, at 68.8 (52.2; 88.5) hours, than in negative BCs (191.7 [181.8; 198.0], p<0.001).

### Treatment adjustments

In order to assess the impact of positive PCR results on the physician's decision to change antimicrobial therapy, all charts were reassessed by an independent infectious diseases consultant and an ICU consultant. In opinion of the arbitrators, 94% of the positive PCR results revealed an important finding which is not suggestive of contamination and should be considered as a true pathogen. In 34% of patients with positive PCR, retrospective evaluation revealed that initial antimicrobial therapy did not cover the retrospectively suspected causative. This included fungi in 4 cases, vancomycin resistant enterococci in 5 cases, and multiresistant staphylococci in 3 cases. The treating physician changed antimicrobial in 38% of the positive PCRs. In patients, where the treating physicians changed antimicrobial therapy because of the PCR result, serum c-reactive protein (CRP) dropped from 219.5 (135.2; 250.8) to 126.0 (104.5; 198.5) mg/l (p = 0.03) within 4 days after change of antimicrobial therapy, and serum procalcitonin (PCT) dropped from 5.5 (2.3; 24.5) to 2.9 (2.0; 8.7) ng/ml (p = 0.01). The arbitrators also assessed that 24.2% of the PCR results would suggest a possible de-escalation.

## Discussion

In this study, we assessed the performance of culture-independent PCR-based detection of pathogens compared with blood culture regarding impact on antimicrobial therapy and turnaround time. We confirmed a significantly higher rate of positivity in PCR than in blood culture but the concordance regarding positive results between culture based methods was poor. In about 34% of the positive PCRs, the initial antimicrobial treatment did not cover the organism detected in the PCR, and was adjusted by the treating physician. Time to positive result was significantly shorter for PCR (PCR 24.2 hours) compared to 68.8 hours for the blood culture but exceeded the frequently suggested 6–8 hours due to limitations in staff availability. The validity of positive PCR results not confirmed by BC was suggested by elevated biomarkers of infection, i.e. C-reactive protein (CRP) and procalcitonin (PCT). In particular PCT has been demonstrated lately to be a good marker for guidance of antibiotic therapy [18].

Our data are consistent with the notion that PCR technology cannot replace blood culture but may add valuable diagnostic information in critically ill patients with suspected sepsis. In this respect, the currently used test (VYOO®, SIRS-Lab GmbH, Jena, Germany) yielded similar findings to those of another assay in a recent multicenter study [8]. In their study, sensitivity to detect culture positive bacteremia was reported to be 0.8 and the specificity was 0.77 for the SeptiFast®-test (Roche Diagnostics). In this study, VYOO® showed a somewhat lower sensitivity while the specificity was similar to SeptiFast®. Likewise, recovery rate of pathogens detected in the blood culture is lower in the VYOO® than in SeptiFast®. One has to take into consideration, however, that comparison of the studies is hampered by the fact that the SeptiFast® study was a clinical study [8] while this study took place under routine clinical conditions.

Early adequate antibiotic therapy is vital in these patients, since both a delay in therapy and inadequate treatment are associated with an increased risk of death [19]–[22]. It has been estimated from prospective interventional trials that at least 10% of septic patients do not receive antibiotics that cover the responsible organism, despite the use of broad spectrum empiric antibiotics [5], [19], [20]. This share may even be higher outside of clinical studies which include recurrent episodes of sepsis with an increase in pathogens that are difficult to cover with empiric antimicrobial therapy. This study thus confirms a relatively high rate of inappropriate antibiotic therapy, and demonstrates that time to readjustment can be significantly shortened by the use of PCR in the clinical setting. This is somewhat supported by the observation that change of antimicrobial therapy resulted in a drop of CRP and PCT. However, this observation needs to be interpreted with caution since these data have been recorded retrospectively and are uncontrolled.

Although the study was not designed to evaluate the impact of early antimicrobial adjustment on patient outcome, our findings point to potential advantages of early adjustment. In 13 patients, the PCR detected a pathogen not covered by the initial antimicrobial therapy, earlier than blood culture or the microbiological specimen from the presumed site of infection. These included patients with multi-resistant pathogens, where even a treatment according to current guidelines is inadequate [5]. Although identification of resistances is still an evolving field in PCR, detection of genes coding resistance to methicillin or vancomycin is possible, and may therefore be helpful in early adjustment of antimicrobial therapy. However, culture based pathogen detection remains the prerequisite for resistance testing so far and can currently not be replaced by the PCR technology. Furthermore, PCR results did not reflect findings from the presumed site of infection.

Some of the PCR results yielded Gram-positive pathogens such as enterococci. Contribution of enterococci to infection is difficult to assess. This is especially true for ICU patients where little data about the involvement of these bacteria are available. Recently, the importance of the involvement of enterococci in severe intraabdominal infections has been documented [23]. Blood cultures positive for enterococci are frequently caused by intraabdominal infections [24]. Nevertheless, the significance of PCR results containing Gram positive bacteria need particularly further investigation.

Changes in antimicrobial therapy also included the addition of antifungal therapy in some cases. The possible contribution of PCR to diagnosing invasive candidiasis has recently been reported [25]. Suspected candidemia according to PCR but not confirmed by BC was associated with a mortality of >80% in a study in which antifungal therapy was not initiated as PCR results were blinded to the treating physician [8]. Furthermore, a prospective randomized trial of patients after stem cell transplantation demonstrated reduced 30 day mortality when the PCR result was taken into account in the clinical decision for antimicrobial therapy [13]. Thus, PCR-based fungal detection seems promising, and should be further investigated in prospective studies addressing antifungal therapy.

Blood cultures are a crucial step in the diagnostic work-up, and are seen as mandatory in septic shock patients [7]. However, blood cultures have several limitations: the rate of positivity strongly depends on the quality of pre-analytic procedures such as sterile puncture, the amount of blood taken, antibiotic pretreatment, and transport conditions [8], [12], [26], [27]. PCR technology is much less vulnerable to these factors, which may be partly responsible for the high rate of positivity also seen here and in other studies [11], [28], [29], including positive results in patients who are already treated with antibiotics [30]. Furthermore, the blood culture results in this study included a high rate of possible skin contaminants. Although true bacteraemia with coagulase-negative staphylococci (CoNS) may be possible due to central venous catheter related infection, endocarditis, prosthetic joint infections, osteomyelitis etc. [31], positive blood cultures with this pathogen are difficult to confirm in the clinical setting. Artificial limitation of positive results by using a cut-off value for CoNS during the analysis of the PCR sample may help in the discrimination between contamination and infection [32]. However, this was not implemented in the used assay. We detected a significant amount of CoNS in the PCR, albeit much less than in the blood cultures. Furthermore, there was no difference regarding markers of inflammation or morbidity between patients with or without CoNS, either in blood culture or PCR.

One of the advantages of PCR is the short time to result compared with culture-based methods. Theoretically, PCR results may be available within 6–8 hours, but this is highly dependent on staff availability. In this study, where PCR was applied in a real clinical setting, median time to result was 23.4 hours on weekdays. Ideal setup conditions could only be reached during weekdays when blood sampling was conducted in the early morning hours. No technician was available to run the PCR at weekends or during the nights. Thus, blood samples drawn on the weekend were analysed the following Monday, resulting in a time to result close to that of blood culture. Therefore, PCR results may be available within one working day only if the laboratory is staffed appropriately. These results need to be taken into account when considering the cost effectiveness and clinical application of PCR technology. In order to reap the full benefits of PCR-based pathogen detection technologies, adjustments to laboratory staffing and workflows might be necessary. Completely automated systems from extraction to read-out although currently not available may overcome this limitation.

Data addressing the clinical value of a PCR result are still scarce [33]. A recent study found that empirical therapy of presumed sepsis needed to be modified in about 8% of cases with positive PCRs [28]. Another study evaluated the initial antimicrobial therapy of patients with malignancies and suspected sepsis by two expert teams where only one team received the PCR results. It was concluded that the PCR result would have improved the empiric antimicrobial therapy in 10% of the patients [13]. These results contrast with the 34% of patients in our study requiring a change of antimicrobial therapy according to the PCR result. Such differences may be explained by various factors, i.e. differences in patient populations. Clearly, the impact of PCR results on the management of antimicrobial therapy needs to be rigorously tested in randomized controlled studies. So far, such degree of evidence has been generated by only the above mentioned trial in patients after stem cell transplantation [13].

Many microorganisms detected in the PCR remained unconfirmed when culture-based pathogen detection was completed. The biological plausibility of PCR results was addressed in a previous study of patients with septic shock. Positive PCR results were associated with higher serum concentrations of biomarkers of inflammation such as interleukin 6 and procalcitonin [8], [27]. The present study confirmed under clinical conditions that a positive PCR is accompanied by elevated serum procalcitonin and CRP levels, supporting the notion that a positive PCR detects a potentially significant event.

Although PCR delivers a higher rate of positivity than blood culture, PCR often remains negative, even in severe sepsis. This is in line with previous studies in this patient population [8], [9], [27]. Reasons for negative PCRs despite a positive blood culture may include contaminants in the blood culture, an incomplete target list, variations in the DNA target, very low rates of pathogen DNA or – depending on the PCR-method – a detection threshold. Therefore, PCR should currently be viewed as an add-on to, rather than as a replacement of culture-based methods. However, there remains a diagnostic uncertainty primarily regarding false negative results. This is reflected in the observed patient cohort by the decision of the physician in charge to continue the initiated therapy (data not shown) despite the PCR result.

Interpretation of the study is limited by its observational nature. Although it was possible to describe potential benefits of including PCR results in the treatment decisions, we cannot conclude that PCR driven treatment decisions improve antimicrobial therapy in a way that infection is controlled earlier or outcome is improved. Such a conclusion would only be possible after a prospective randomized trial.

In conclusion, the results of this observational study support the hypothesis that PCR results are available faster, are more frequently positive, and may result in earlier adjustment of antimicrobial therapy. However, our data point to the fact that this shorter time to result can only be fully exploited when the laboratory is adequately staffed for a 24 hour/7 day service, or when point of care systems become available. Due to its potential to improve outcome of patients with life-threatening infection, the clinical utility and cost effectiveness of PCR should now be tested in adequately powered randomized controlled clinical trials.
